# Acceptance and Commitment Therapy versus Social Support for Smoking Cessation for People with Schizophrenia: A Randomised Controlled Trial

**DOI:** 10.3390/jcm10194304

**Published:** 2021-09-22

**Authors:** Yim-Wah Mak, Alice-Yuen Loke, Doris Y. P. Leung

**Affiliations:** School of Nursing, The Hong Kong Polytechnic University, Hong Kong, China; alice.yuen.loke@polyu.edu.hk (A.-Y.L.); doris.yp.leung@polyu.edu.hk (D.Y.P.L.)

**Keywords:** schizophrenia, smoking cessation, Acceptance and Commitment Therapy, experiential avoidance, emotion regulation

## Abstract

Smoking is prevalent among people with schizophrenia. It has been found that Acceptance and commitment therapy (ACT) is effective for treating psychotic symptoms and addictive behaviours, but the therapy has not been modified to help individuals with schizophrenia to quit smoking. A randomised controlled trial was conducted with the objective of comparing a 10-week, individual, face-to-face ACT programme (*n* = 65) to a social support programme on smoking cessation, experiential avoidance, and emotion-regulation strategies among people with schizophrenia who smoke (*n* = 65). The primary outcome was self-reported smoking abstinence for 7 days at 6 months after the start of the intervention. Secondary outcomes were self-reported and biochemically validated quit rates post-intervention. The Avoidance and Inflexibility Scale (AIS), Acceptance and Action Questionnaire II (AAQII), and Emotion Regulation Questionnaire (ERQ) were employed. The self-reported quit rates in the ACT group were higher than in the social support group, although no significant differences were found (6 months: 12.3% vs. 7.7%, *p* = 0.56, 12 months: 10.8% vs. 7.7%, *p* = 0.76). We found significantly greater improvements in smoking-specific and ACT-specific experiential avoidance and less reliance on emotion regulation strategies in the ACT group at some time points. Overall, ACT is better than social support at enhancing experiential avoidance and reducing reliance on emotion regulation strategies in adults with schizophrenia who smoke. However, ACT did not produce a much better result than social support in helping them to completely quit smoking.

## 1. Introduction

Evidence that smoking cigarettes causes fatal diseases and leads to death is well established [1]. Past studies have found that smokers with mental disorders die an average of 25 years sooner than the general population [2]. Amongst people with mental disorders, the prevalence of smoking has been reported to be highest among people with schizophrenia, ranging from 54% to 90% [3,4]. This is more than two to three times higher than that of the general population [5]. Moreover, cessation rates among people with schizophrenia are very low [6,7]. Patients with mental health problems often use cigarettes to self-medicate to compensate for deficits associated with the illness, antipsychotic medications, or both [8]. This elevated smoking rate is a potential cause of the increased risk of premature death due to medical illnesses, including cardiovascular diseases and cancers, among people with schizophrenia [9]. In addition, psychiatric patients with a smoking habit require a larger dose of drugs to control the symptoms of psychosis than those without a smoking habit [10].

Efficacious pharmacological and non-pharmacological smoking cessation interventions are available for the general population [11] and for people with serious mental illnesses [12], but efforts to assist people with schizophrenia to change their smoking habits have been severely lacking [7]. Although bupropion has been found to be efficacious for use in smoking cessation among people with schizophrenia [7], the evidence for sustained abstinence was based on five small trials conducted by two research groups with a total sample size of 214. Furthermore, bupropion is not recommended for use by smokers who are receiving treatment for neuropsychiatric problems or who are on antidepressants [13]. Side-effects such as dry mouth [14], jitteriness, light-headedness, muscle stiffness, frequent nocturia, and decreased concentration [15] have been reported. There have also been reports of the recurrence of psychotic symptoms [16] and allergic reactions to the medication [17]. These side-effects have hindered efforts by the participants to adhere to a regime of taking the medication [18].

A few clinical trials have been conducted to examine the efficacy of behavioural interventions for smoking cessation in this specific group. Meta-analytic data, including those from the abovementioned five trials, indicate that those interventions were of no benefit in either the medium or long term [19]. The behavioural interventions in these trials adopted smoking cessation counselling programmes on motivational interviewing and/or cognitive behavioural therapy that demonstrated effectiveness in the general smoking population. Individuals with schizophrenia may find it hard to control or change their irrational thoughts because of symptoms such as hallucinations, delusions, or racing thoughts, as these seem very real to them. Cultivating a willingness among such individuals to experience all thoughts and feelings more mindfully may be easier to achieve. It might be helpful to develop behavioural smoking cessation interventions that target specific aspects of schizophrenia, such as interventions that are tolerant of distress and set flexible goals on smoking cessation, for individuals who smoke and have been diagnosed with schizophrenia [20].

Acceptance and Commitment Therapy (ACT) has shown promise for the treatment of a variety of psychological disorders, including psychosis, anxiety and stress, depression, and substance abuse [21]. From the ACT perspective, all of these disorders functionally share a common feature: the attempt by individuals to control or reduce private aversive events (e.g., anxiety, sadness, cravings, and psychogenic pain). The general goal of ACT is to increase psychological flexibility, which is the ability to more fully connect with the present moment, and to change or persist in a particular behaviour when doing so serves valued ends. ACT incorporates acceptance, mindfulness, and the clarification of values to enhance traditional behavioural interventions. It focuses on modifying the individual’s relationship to his or her thinking. It encourages individuals to accept and experience internal events non-judgementally (i.e., mindfully), while simultaneously working toward the pursuit of personally defined behavioural goals [22]. Clients are encouraged to minimise strategies aimed at over-controlling unpleasant private experiences, and instead to notice non-judgementally the occurrence of negative thoughts, feelings, and sensations that may impede the attainment of goals without acting on them. It has recently been argued that ACT is a more feasible, acceptable, and potentially efficacious intervention for smoking cessation than traditional behavioural interventions [23]. However, there has been no randomised control trial examining the efficacy of ACT for smoking cessation targeting people with schizophrenia, although such a study is one of the priorities for advancing research in this area [20].

On the other hand, social support has been highlighted as a key component in smoking cessation treatments for people with schizophrenia. A qualitative study revealed that people with schizophrenia were fearful of being alone, relapsing into a mentally unstable condition, and reintegrating into the community. They often smoked to deal with their psychological distress and cope with nerves and used smoking as a means of gathering socially [24]. A mixed-methods study reported that both people with schizophrenia and healthcare providers endorsed the idea that smoking cessation interventions should include sessions on social support [25].

Thus, the aim of the current study was to evaluate the efficacy of ACT vs. social support for smoking cessation in a sample of smokers with schizophrenia who reside in community-based residential settings. We hypothesised that, after the intervention, participants in the ACT group would exhibit (1) greater improvement in (i) smoking abstinence rate, (ii) smoking reduction rate (at least 50%), and (iii) quit attempt rate; (2) a reduction in (i) smoking-related experiential avoidance and (ii) smoking-specific and ACT-specific experiential avoidance; and (3) less reliance on strategies to regulate emotions. In addition, we assessed the acceptability of the ACT and social support interventions among individuals with schizophrenia who smoke.

## 2. Materials and Methods

### 2.1. Study Design

We conducted a prospective, double-blinded, individually randomised, controlled, parallel trial, which was registered on the clinical trial website (https://clinicaltrials.gov: NCT03253445). Randomisation was carried out according to a code generated by the computer, a block size of six with a 1:1 allocation. The random numbers for the group assignment were then put in sequentially ordered, opaque, sealed envelopes, which were prepared by a member of the project team who had had no contact with the subjects prior to their recruitment. Both the participants and assessors were blinded to the group allocation. The study was conducted in accordance with the CONSORT ethical guideline. All of the participants gave their written informed consent to participate in the study, and the study was approved by the Ethics Committee of the Hong Kong Polytechnic University.

A power analysis of the study was performed based on the primary outcome, the self-reported 7-day prevalence quit rate at 6 months. The number of proposed subjects in each treatment group was 78, which allowed for a power of 80% to detect a difference of 11.9% between the two groups [26].

### 2.2. Participants and Setting

The study population consisted of current smokers who had been diagnosed with schizophrenia and were living in one of the 51 community-based residential mental health rehabilitation settings in Hong Kong. We included participants if they were at least 18 years old, had smoked at least one cigarette daily in the past 30 days, and were able to communicate in Cantonese. We excluded those with disorientation, developmental disabilities, and/or an organic mental disorder, who had received a diagnosis of alcohol or drug dependence in the year preceding recruitment, whose medication regime had been revised in the last 3 months, or who were currently participating in a smoking cessation programme. For the general population, greater nicotine dependence as measured by the time of the first cigarette and the number of cigarettes smoked per day is the most consistent predictor of smoking cessation failure [27,28]. However, there is a lack of similar studies on patients with schizophrenia. In addition, previous research reports have stated that most people become daily smokers after trying one cigarette [29]. In order to help everyone quit smoking, we did not exclude participants based on the number of cigarettes that they consumed.

All patients with schizophrenia who smoked were referred by staff of the residential settings run by non-governmental organisations and private sector groups to meet our research assistant in a specific room in the residential rehabilitation centre for screening, and eligible patients were invited to take part in the trial. Recruitment was conducted over a period of about 1.5 years between May 2015 and December 2016. The trial was terminated due to the lack of resources.

### 2.3. Treatment Conditions and Procedure

#### 2.3.1. Acceptance and Commitment Therapy

Participants in both groups received the usual care consisting of a 5-min brief educational talk encouraging smoking cessation plus a self-help written leaflet on smoking cessation. In addition to the usual care, all of the participants in the Intervention Group (IG) were offered 10 weekly face-to-face individual sessions of ACT by a trained therapist. Each session lasted for about 30 min. Most components of the ACT intervention in this study were drawn from an ACT manual developed by the ACT founder and his team [30], while specific metaphors for smoking cessation were extracted from relevant studies [26,31]. Based on previous studies [24,32], some metaphors and exercises were modified to fit the Chinese target group. The intervention was an integration of psycho-education and the six core ACT processes (acceptance, present, values, committed action, self as context, and defusion). The aim was to help the participants to (1) increase their acceptance of thoughts and feelings related to cigarette cravings and withdrawal symptoms; (2) clarify their life values and enhance engagement with those values; and (3) build up patterns of values to which they are committed and which they can identify even in the presence of cravings and/or withdrawal symptoms. Each session that was delivered was heavily experiential in nature. Apart from being asked to actively engage in conversation and mindfulness exercises, the participants were also encouraged to practise home assignments between sessions. Supplementary Table S1 gives an outline of the sessions and their contents.

#### 2.3.2. Social Support

As a comparison intervention, social support as the most convenient standard intervention for smoking cessation was chosen. We provided a brief educational talk of about 5 min encouraging the participants to quit smoking, as well as a self-help leaflet on smoking cessation. Ten sessions of face-to-face social support were provided on a weekly basis. Each session lasted for about 5–10 min. Social support was chosen because a systematic review showed that interventions that improve social support for smoking cessation may be of greater importance to disadvantaged groups than to other groups, as disadvantaged people experience fewer opportunities to informally access such support [33]. The participants were guided by the therapist to share their views on topics related to smoking, such as their smoking habits, cravings, and cessation experiences, and their intention to reduce their consumption of tobacco. The objective was to create an atmosphere of social support with the intention of delivering a tone of acceptance, concern, and allowance of whatever the participant had been experiencing relating to smoking.

#### 2.3.3. Treatment Integrity

The ACT supervisor of this study (first author) has more than 20 years of experience in health counselling, particularly smoking cessation counselling, and has received training in the principles of ACT developed by Hayes and his team [30]. The ACT facilitator was asked to complete a self-competency checklist related to the principles and practices of adopting ACT in smoking cessation. Throughout the study, the ACT skills of the facilitator were supervised by the ACT supervisor. Three strategies were used to enhance treatment integrity: (1) continuous training of the ACT intervention in smoking cessation was provided to equip the ACT facilitator until three consecutive sessions were delivered that met the criteria before the ACT facilitator delivered the ACT sessions on his/her own; (2) on a bi-weekly basis, the ACT supervisor met with the ACT facilitator to provide support and to discuss questions or issues about smoking cessation using ACT throughout the whole study; and (3) to monitor the quality of the intervention, 20% of the subjects were randomly selected to be recorded for at least one session. Each recording was rated by two independent reviewers: the ACT supervisor and a peer ACT facilitator who was working on another research project. Each of the six core processes of the ACT intervention was assessed based on the principles of ACT as stated in the fidelity form [34]. A 7-point Likert adherence scale, from never true to always true, was used to assess the level of competency of the ACT facilitator. To pass the competency test, the ACT facilitator needed to receive a rating of 6/7 on 80% of the assessment items. Any discrepancies from the fidelity standard that were identified were discussed with the facilitator for modification. Support and further training were provided when needed.

### 2.4. Assessments

Four assessment sessions were planned: immediately after obtaining consent (baseline), a post-treatment assessment at 3 months after the initial contact, and two follow-up assessments at 6 and 12 months. The pre- and post-treatment assessments were conducted by research assistants who were not involved in the treatment and were blinded to the treatment conditions. All assessments were conducted at the residential centres that the participants were attending.

### 2.5. Measures

The primary outcome measure was self-reported 7-day point prevalence tobacco abstinence at 6 months after the initial contact. Subjects who reported that they had not smoked a cigarette in the past 7 days were considered to be self-reported quitters. Although underreporting due to social desirability is possible, self-reported smoking was found to have good validity in our previous study among the Hong Kong population [35].

Secondary outcomes included self-reported 7-day point prevalence tobacco abstinence at 12 months, biochemically validated tobacco abstinence at 6 and 12 months, having made at least one attempt at quitting smoking for at least 24 h at some point prior to the interview at 6 and 12 months (quit attempt rate), and a reduction in cigarette consumption by at least 50% compared to baseline at 6 and 12 months. Except for biochemically validated tobacco abstinence at 12 months, all of the outcomes were measured based on self-reports. Those who were lost to follow-up were treated as smokers, as having made no attempts to quit, and as non-reducers. Subjects who reported that they had stopped smoking for at least 7 days were invited to undergo measurements of their CO and urinary cotinine levels. Subjects were regarded as validated quitters if they had a urinary cotinine concentration of ≤115 ng/mL or an exhaled CO level of <6 ppm [36]. Those who were lost to follow-up or who refused to participate in validation tests were treated as smokers.

Three process measures were included in the study, measured at each assessment time point.

- Smoking-related experiential avoidance was measured using the Avoidance and Inflexibility Scale (AIS) [26]. The AIS consists of 13 items, rated from 1 (“not at all”) to 5 (“very likely”), which evaluate the smoker’s endorsement of avoidance strategies in relation to smoking and smoking cessation. Higher scores indicate greater levels of inflexibility and avoidance in the presence of difficult thoughts, feelings, and sensations related to smoking. Higher scores indicate an individual’s engagement in strategies to avoid internal cues such as negative thoughts, feelings, and sensations, and with inflexible behaviours such as smoking whenever a negative thought/feeling/stress is experienced;
- ACT-specific experiential avoidance was measured using the 10-item Acceptance and Action Questionnaire II (AAQII) [37]. The AAQII provides a broad measure of a person’s experiences with avoidance, immobility, acceptance, and action. Items were rated on a 4-point Likert scale from 1 (“strongly disagree”) to 7 (“strongly agree”), with higher scores indicating a greater level of psychological flexibility;
- Emotion regulation strategies were measured using the Emotional Regulation Questionnaire (ERQ) [38], which consists of 10 items measuring the respondents’ tendency to regulate their emotions in two ways: through cognitive reappraisal and the suppression of expressions. Items were rated on a 7-point Likert scale from 1 (“strongly disagree”) to 7 (“strongly agree”), with higher scores indicating greater levels of endorsement in the particular manner of regulation.

### 2.6. Statistical Analysis

Study hypotheses were evaluated using intention-to-treat analyses, including all participants enrolled and randomised to each condition. Baseline group differences by treatment group were examined using independent samples t-tests for continuous variables and chi-squared tests for categorical variables. For the binary outcomes, we used χ^2^ tests to assess the effect of the intervention and calculated the absolute difference in percentages with a 95% confidence interval (CI). For continuous measures, generalised estimating equation (GEE) models, with the attendance rate in the 10-session treatment as a covariate, were used to assess treatment processes from baseline to the follow-up assessments. GEE models accounted for the dependence between repeated measures within individuals and can take into account missing data [39]. The interactions with each time variable of the treatment groups were assessed to examine whether the groups differed significantly with regard to changes in outcomes. All of the patients were included in the analysis based on the intention-to-treat principle whenever applicable. Analyses were performed using SPSS version 25.0 (IBM, Armonk, NY, USA).

## 3. Results

### 3.1. Participant Flow and Sample Description

Of the 235 eligible patients, 130 were enrolled and randomised, with 65 participants in each of the ACT and social support groups. There was no significant difference in attrition between the two groups at each assessment time point (3 months: 7.7% vs. 4.6%, *p* = 0.46; 6 months: 12.3% vs. 15.4%, *p* = 0.24; 12 months: 18.5% vs. 18.5%, *p* = 1). The participants in both groups had similar attendance rates at their allocated treatment programme (0.84 ± 0.26 vs. 0.84 ± 0.25, *p* = 0.68). The study flowchart is available in supplementary Figure S1.

Table 1 shows the baseline demographic and smoking behaviours for each treatment group. Most of the participants were male (*n* = 112, 86%) and had attained a secondary education (*n* = 88, 68%). The sample distribution contained a high percentage of smokers who had never attempted to quit smoking before (*n* = 73, 56%) and were not prepared to quit smoking within one month of the initial contact (*n* = 79, 61%) when compared to populations typically recruited to take part in smoking studies in Hong Kong [35,40]. Participants in the ACT and social support groups did not differ significantly in any of the baseline variables, with the exception of participants in the social support group, who reported a significantly higher level of perceived social support from friends (*p* = 0.04).

### 3.2. Effect of ACT on Binary Smoking-Related Measures

The results of the smoking-cessation measures are presented in Table 2. We observed a trend of difference in self-reported 7-day point prevalence at the 6-month (primary outcome) and 12-month follow-ups. The 12.3% and 10.8% rates of abstinence in the intervention group were greater than the 7.7% and 7.7% rates for the control group, although no statistically significant differences between the groups were observed. A similar trend of difference in the biochemically validated quit rates was also found in the 6-month and 12-month follow-ups. The 9.2% and 9.2% rates of biochemically validated abstinence in the intervention group were greater than the 4.6% and 6.2% rates for the control group, while the self-reported smoking reduction and quit attempt rates were slightly lower in the ACT group than in the social support group at both the 6- and 12-month follow-ups.

### 3.3. Effect of ACT on Exhaled CO Level and Process Measures

Table 3 presents the GEE results on the exhaled CO level and the three process measures. The exhaled CO level was reduced in the ACT group and was increased slightly in the social support group over the study period, and the difference in the change in the exhaled CO level was significant at 12 months (*p* = 0.03). Significant group differences in rates of change in all three process measures were observed in a number of assessment time points. For smoking-specific experiential avoidance, participants in the ACT group reported a gradual decrease in their AIS scores from baseline to the 12-month follow-up, while participants in the social support group showed a trend of increase. Significant group differences were observed at 6 months (*p* = 0.02) and 12 months (*p* = 0.04).

With regard to ACT-specific experiential avoidance, there was a slight increase from baseline to 3 months among participants in the ACT group, and then a sharp decrease in AAQII afterwards, whereas there was a gradual increase in AAQII among the social support group over the study period. A significant between-group difference in the change in AAQII scores was observed at 12 months (*p* = 0.03).

For emotional regulation, the ACT group reported a significantly greater reduction in their level of endorsement of both the strategies of cognitive reappraisal (*p* = 0.04) and suppression (*p* = 0.002) than did the social support group from baseline to 3 months. Participants in the two groups did not differ significantly in their level of endorsement of cognitive reappraisal and suppression for emotional regulation at the both 6- and 12-month follow-ups. There were no reports of any adverse effects from the two interventions.

### 3.4. Acceptaibility of the Intervention

The results on the acceptability of the study as perceived by the participants are presented in Table 4. Comparing opinions collected from participants of the ACT and social support interventions at the 12-month follow-up interviews, both groups expressed a similar level of satisfaction with the smoking cessation counselling approaches to which they were assigned. Most said that they were satisfied or very satisfied with the face-to-face approach taken in their assigned smoking cessation intervention and felt comfortable and natural during the conversation with the counsellor; with the arrangement where the counsellor personally goes to the institution or workshop for counselling every week; and with the counselling at the place of their residential institution. Most agreed that if a staff member had not personally introduced the counselling programme to them, they might not have been exposed to this intervention service. After participating in this programme, most said that their intention to quit smoking had increased, that they had tried to quit smoking or to reduce their level of smoking and would introduce this plan to friends who smoked.

## 4. Discussion

This is the first report of a randomised controlled trial in which ACT was used to help smokers with schizophrenia to quit smoking. Thus, the findings of the present study are an important addition to smoking cessation studies involving an under-represented segment of the population. One strength of this trial is that it reached one of the most vulnerable groups of smokers—those who have been diagnosed with schizophrenia—who also have one of the highest rates of prevalence of smoking. Another is that abstinence was rigorously measured, through both self-reports and biochemical verification.

The present study found that ACT led to a reduction in avoidance and inflexibility, but not to an increase in the success rates for smoking cessation. Nevertheless, one significant achievement in the use of Acceptance and Commitment Therapy was the enhanced psychological flexibility of the participants assigned to the ACT group. This process of identifying avoidance behaviour and developing a more flexible approach to addressing it is central to this intervention and is regarded as an important process indicator supporting the use of ACT for making behavioural changes [41]. Compared to the very limited studies conducted in the past on smoking cessation among individuals who had been diagnosed with schizophrenia, which involved a follow-up period of longer than 6 months, the quit rate of the ACT group in the present study at 6 months (12.3%) was comparable to that in the study on the use of bupropion (18.8%; [13]) and higher than that reported in Evins (4%; [17]) and in the study in which bupropion was used together with CBT (11%; [42]). The present study employed only a single behavioural intervention; however, previous studies found that a combination of smoking cessation medications such as nicotine, vareniciline, and bupropion [43] and a behavioural intervention would yield significantly better quit rates when compared with using behavioural interventions only [44]. Thus, it could be possible that solely employing an ACT intervention would be insufficient to help smokers successfully quit smoking. In future studies, consideration could be given to combining cessation medications with behavioural interventions to help this special group of people. These components should be tested separately to confirm which, if any, of them would be important factors in helping people to successfully quit smoking, to determine its effects on the required dose of medications to control psychotic symptoms, and to assess whether the components or a combination of them would be acceptable to patients.

Furthermore, the intervention completion rate (84%) for both groups was comparable to that for the general population of a smoking cessation intervention study conducted in Hong Kong (given printed self-help quitting materials—442/485 = 91% vs. stage-matched counselling—396/467 = 85%). The attrition rate (13.8%) at the 6-month follow-up in the present study was also comparable to that for the general population (12%) in Hong Kong [45], and the attrition rate was far lower than is usual among disadvantaged populations [46]. The high completion rate of the interventions and the low attrition rates for both groups can probably be attributed to the fact that the deliverables of the interventions were highly acceptable to the participants. The engagement of patients is one of the most common challenges for clinicians using psychotherapies to help people with mental illness [47]. The effectiveness of the intervention would certainly be affected if patients failed to complete the whole course of the intervention. The discontinuation of the therapy may be related to the limited immersive power of the intervention [48]. Therefore, metaphors and mindfulness were used in the ACT intervention because they help to make concepts appear more tangible to the participants. The participants were thus willing to experience thoughts and feelings related to smoking and quitting, complete the intervention, and recommend the intervention to other people. Thus, ACT would be an alternative to traditional psychotherapies for helping people with schizophrenia.

There are several possible reasons why the ACT intervention did not outperform the social support intervention for smoking cessation. First, we could not reach the original estimated sample size of 150, which led to the study being underpowered, although we tried hard to obtain support from 51 eligible community settings when recruiting subjects. Nevertheless, for future studies our study has provided an estimate of the size of the effect of an ACT intervention compared to that of social support in helping this vulnerable group to quit smoking. Second, according to a systematic review of eight studies on peer support interventions for smoking cessation in disadvantaged groups [49], social support is emerging as a highly effective and empowering way for people to manage health issues. Many participants in the present study perceived that they received only moderate support from family and friends. The participants in the social support group had reported significantly higher perceived support from friends before the intervention started, which may partly explain the null effect on the differences in smoking cessation rates between the ACT group and the social support group, as social support was very important to this group of individuals [50]. Third, the intervention dose of 10 face-to-face sessions might have been insufficient to develop the ability of people with schizophrenia who smoke to address their physiological, psychological, and social challenges. We found that the participants in the present study could be characterised as “hard-core smokers” [51]. For example, at baseline 48% (*n* = 63) of the 130 participants were at the pre-contemplation stage of quitting, but none had started quitting (taking action to quit) at that time, while the corresponding figures for the general population of Hong Kong Chinese were 15.6% and 3.7% [40]. Studies have found that hard-core smokers are the least likely group of people to quit smoking [52]. The number of hard-core smokers in this study might have been larger than our estimates suggested. Likewise, when comparing the smoking history of the participants in this study with that of the general population, the participants in this study were found to have averaged more years of smoking, and only a few had previously attempted to quit [46]. As treatment intensity is associated with cessation [53], the participants in the present study might have needed more time to prepare to quit smoking. Fourth, behavioural counselling alone might not be enough to help this population to completely quit smoking. For future studies, social support [25] or Nicotine Replacement Therapy [53] could supplement an ACT intervention. Furthermore, other factors associated with smoking, such as a dependence on other substances [54,55], states of mood, other psychiatric status, and the existence of a tobacco restriction policy may also affect the success of an individual’s smoking cessation effort. Further investigations with a higher intervention dose and involving an effort to determine whether the participants were consuming other substances such as alcohol or drugs would strengthen our results, but the data have yet to be collected in the present study.

There are several limitations to the present study, so the results should be viewed with caution. First, the final number of participants did not reach the target sample size of 156, making the study underpowered. Second, we did not record the length of treatment in the social support group, which might have been more intensive than designed. Third, the participants were recruited from many residential sites in the community. Although this was positive for the generalisability (external validity) of the study, the sample may have been too heterogeneous in that the participants would have been drawn from facilities that might have had different policies on the enforcement of smoke-free settings, which might have affected the outcome of the study. Fourth, previous studies found that increased frequency of cognitive reappraisal and reduced frequency of expressive suppression would have better effects on the regulation of emotions and lead to more favourable smoking cessation outcomes [56,57]. In the present study, when compared with participants allocated to the social support group, participants who completed ACT used both cognitive reappraisal and expressive suppression less frequently to regulate their emotions at 3 months after the intervention. The difference was not observed at the 6th and 12th month follow-ups. Thus, it is not certain that the effects of ACT on enhancing the emotion regulation of individuals with schizophrenia who smoke have any impact on the smoking cessation outcomes. Further studies are warranted to address the above limitations, with a bigger sample size to investigate the effects of ACT for smoking cessation among individuals with schizophrenia.

## Figures and Tables

**Table 1:** Baseline characteristics of the participants (*n* = 130).

	ACT (*n* = 65)	Social Support (*n* = 65)	*p*-Value
Gender, *n* (%)			0.13
Male	59 (90.8)	53 (81.5)	
Female	6 (9.2)	12 (18.5)	
Age, M ± SD	49.7 ± 11.3	50.0 ± 12.0	0.88
Educational attainment, *n* (%)			0.08
Primary or below	19 (29.2)	15 (23.1)	
Secondary	45 (69.2)	43 (66.2)	
Tertiary or above	1 (1.5)	7 (10.8)	
Marital status, *n* (%)			0.76
Single	43 (66.2)	41 (63.1)	
Married	14 (21.5)	13 (20.0)	
Divorced/Separated/Widowed	8 (12.3)	11 (16.9)	
Years of onset of schizophrenia ^1^, M ± SD	19.9 ± 13.6	20.9 ± 12.6	0.68
Years of smoking, M ± SD	27.6 ± 13.5	28.3 ± 13.6	0.75
Daily cigarette consumption, M ± SD	11.2 ± 7.7	13.5 ± 9.7	0.15
Pack year ^2^, M ± SD	14.7 ± 13.5	19.4 ± 15.8	0.07
Fagerstrom nicotine dependence ^3^, M ± SD	3.7 ± 2.1	4.2 ± 2.4	0.29
Made previous quit attempts, *n* (%)			0.60
Yes	30 (46.2)	27 (41.5)	
No	35 (53.8)	38 (58.5)	
Nicotine withdrawal symptoms ^4^, M ± SD	10.2 ± 8.3	13.3 ± 8.3	0.16
Stages of change, *n* (%)			0.24
Pre-contemplation	27 (41.5)	36 (55.4)	
Contemplation	8 (12.3)	8 (12.3)	
Preparation	30 (46.2)	21 (32.3)	
Perceived social support ^5^, M ± SD			
Family	6.2 ± 1.8	6.3 ± 2.0	0.79
Friends	6.3 ± 1.9	7.1 ± 2.3	0.04

M = mean; SD = standard derivation. ^1^ Intervention group (*n* = 57), control group (*n* = 58). ^2^ It is calculated by multiplying the number of packs of cigarettes smoked per day by the number of years the person has smoked. ^3^ Range 0–6, with higher scores indicating more nicotine dependence. ^4^ Intervention group (*n* = 30), control group (*n* = 27); higher scores indicate more nicotine withdrawal symptoms. ^5^ Range 4–12, with higher scores indicating less perceived social support.

**Table 2:** Abstinence from cigarette smoking, reduction in smoking at the 6- and 12-month follow-ups and quit attempts within the past 12 months.

	ACT (*n* = 65) *n* (%)	Social Support (*n* = 65) *n* (%)	Absolute Difference (95% CI)	*p*-Value
Primary outcome at 6 months				
Self-reported 7-day prevalence quit rate at 6 months	8 (12.3)	5 (7.7)	−0.05 (−0.15–0.06)	0.56
Secondary outcomes				
Self-reported 7-day prevalence quit rate at 12 months	7 (10.8)	5 (7.7)	−0.03 (−0.13–0.07)	0.76
Biochemically validated quit rate at 6 months	6 (9.2)	3 (4.6)	−0.05 (−0.13–0.04)	0.49
Biochemically validated quit rate at 12 months	6 (9.2)	4 (6.2)	−0.03 (−0.12–0.06)	0.74
Self-reported reduction in smoking ≥50% at 6 months	21 (32.3)	23 (35.4)	−0.03 (−0.13–0.19)	0.85
Self-reported reduction in smoking ≥50% at 12 months	24 (36.9)	23 (35.4)	−0.02 (−0.18–0.15)	1.00
Had stopped smoking for at least 24 hr at some point prior to the interview at 6 months	17 (26.2)	20 (30.8)	−0.05 (−0.11–0.20)	0.70
Had stopped smoking for at least 24 hr at some point prior to the interview at 12 months	19 (29.2)	29 (44.6)	−0.15 (−0.01–0.32)	0.10

**Table 3:** GEE results on changes in process measures over time by groups.

	ACT (*n* = 65) Estimated Mean ± SE ^6^	Social Support (*n* = 65) Estimated Mean ± SE ^6^	Between-Group Difference from Baseline (95% CI)	*p*-Value
Exhaled CO level ^1^				
Baseline	16.97 ± 2.20	15.76 ± 2.04		
6 months	14.41 ± 2.20	15.90 ± 2.12	−2.71 (−7.39, 1.98)	0.26
12 months	11.95 ± 2.31	16.66 ± 2.10	−5.92 (−11.21, −0.64)	0.03
AIS ^2^				
Baseline	41.18 ± 1.21	38.51 ± 1.21		
3 months	40.63 ± 1.37	40.47 ± 1.10	−2.51 (−6.10, 1.08)	0.17
6 months	38.90 ± 1.31	41.84 ± 1.37	−5.61 (−10.38, −0.84)	0.02
12 months	38.49 ± 1.76	41.12 ± 1.43	−5.30 (−10.25, −0.35)	0.04
AAQII ^3^				
Baseline	28.66 ± 1.23	29.74 ± 1.23		
3 months	30.81 ± 1.38	29.24 ± 1.11	2.65 (−1.21, 6.51)	0.18
6 months	25.96 ± 1.28	28.19 ± 1.27	−1.15 (−5.58, 3.28)	0.61
12 months	21.98 ± 1.28	27.77 ± 1.42	−4.70 (−8.95, −0.46)	0.03
ERQ-Cognitive Reappraisal ^4^				
Baseline	18.11 ± 1.19	18.88 ± 0.98		
3 months	14.66 ± 0.92	19.00 ± 0.83	−3.57 (−6.92, −0.23)	0.04
6 months	19.94 ± 1.00	19.40 ± 1.10	1.31 (−2.12, 4.73)	0.45
12 months	18.11 ± 1.48	18.07 ± 1.11	0.80 (−3.21, 4.81)	0.70
ERQ-Suppression ^5^				
Baseline	12.80 ± 0.77	13.92 ± 0.77		
3 months	10.19 ± 0.70	14.95 ± 0.66	−3.63 (−5.94, −1.33)	0.002
6 months	13.46 ± 0.77	13.76 ± 0.79	0.83 (−1.92, 3.58)	0.55
12 months	14.75 ± 0.82	13.02 ± 0.81	2.85 (−0.27, 5.98)	0.07

^1^ ACT (*n* = 61), Social Support (*n* = 65). ^2^ Avoidance and Inflexibility Scale. ^3^ Acceptance and Action Questionnaire II. ^4^ Emotional Regulation Questionnaire—Cognitive Reappraisal subscale. ^5^ Emotional Regulation Questionnaire-Suppression subscale. ^6^ standard error.

**Table 4:** Levels of satisfaction with the counselling.

		Very Agree	Agree	Disagree	Strongly Disagree	*p*
I am satisfied with having this smoking cessation programme via face-to-face counselling	Intervention	15 (31.9%)	31 (66%)	1 (2.1%)	-	0.84
	Control	21 (37.5%)	34 (60.7%)	1 (1.8%)	-	
I feel comfortable and natural during the conversation with the counsellor.	Intervention	13 (27.7%)	31 (66%)	3 (6.4%)	-	0.43
	Control	22 (39.3%)	30 (53.6%)	4 (7.1%)	-	
I am satisfied with the arrangement where the counsellor personally goes to the hostel or workshop for counselling every week.	Intervention	14 (29.8%)	29 (61.7%)	4 (8.5%)	-	0.80
	Control	19 (33.9%)	31 (55.4%)	6 (10.7%)	-	
I am satisfied with the location of the counselling that I received in the hostel or workshop.	Intervention	11 (24.4%)	33 (73.3%)	1 (2.2%)	-	0.30
	Control	20 (39.2%)	30 (58.8%)	1 (2.0%)	-	
If the staff member had not personally introduced the counselling programme to me at this hostel, I might not have been exposed to this service.	Intervention	11 (24.4%)	32 (71.1%)	1 (2.2%)	1 (2.2%)	0.45
	Control	20 (40%)	28 (56%)	1 (1.5%)	1 (1.5%)	
After participating in this programme, my intention to quit smoking increased.	Intervention	9 (19.1%)	28 (59.6%)	10 (21.3%)	-	0.07
	Control	23 (41.1%)	25 (44.6%)	7 (12.5%)	1 (1.8%)	
After participating in this programme, I have tried to quit smoking or reduce my smoking.	Intervention	9 (19.1%)	33 (70.2%)	5 (10.6%)	-	0.03
	Control	22 (39.3%)	25 (44.6%)	6 (10.7%)	3 (4.6%)	
Would you introduce this programme to other roommates or coworkers who have a smoking habit?	Intervention	13 (27.7%)	32 (68.1%)	2 (4.3%)	-	0.38
	Control	23 (41.1%)	30 (53.6%)	2 (3.6%)	1 (1.8%)	

## Supplementary Materials

The following are available online at https://www.mdpi.com/article/10.3390/jcm10194304/s1, Figure S1: study flowchart, Table S1: outline of the Acceptance and Commitment Therapy sessions for smoking cessation.
