# Supplementary material for: Acceptance and Commitment Therapy versus Social Support for Smoking Cessation for People with Schizophrenia: A Randomised Controlled Trial
Source: J Clin Med. 2021 Sep 22;10(19):4304. doi: 10.3390/jcm10194304 (PMC8509331; doi:10.3390/jcm10194304)

Table S1 Outline of the ACT sessions for smoking cessation

Session	ACT Processes	Session plan
1	Values	-Explore the patient's values related to smoking cessation.<br>-Identify treatment goals and link them to the values.<br>-Support the patient's goal of either no smoking or reduced and controlled amounts of smoking.
	Acceptance	-Identify the distinction between smoking and urges to smoke.
2	Acceptance	-Discuss the short-term versus long-term effectiveness of attempts to control urges.<br>-Identify the negative impacts of attempts to control urges.<br>-Highlight the paradoxical nature of attempts to control urges, using the Person in the Hole metaphor.<br>-Reinforce the futility of attempts to control urges.<br>-Identify attempts to control urges as part of the problem, using the pink elephant and dim sum exercise
3	Acceptance	-Introduce acceptance as an alternative to control, using the tug of war with a monster metaphor.<br>-Review acceptance by demonstrating that the willingness to experience urges is a chosen behaviour and an alternative to control, using the tug of war with a monster metaphor.<br>-Identify the decrease in effort required to willingly experience urges.
	Values	-Briefly discuss the patient's values to give purpose and meaning to acceptance.<br>-Discuss what could be gained by letting go of the control agenda.
	Committed action	-Make behavioural commitments to gradually reduce smoking.
4-7	Defusion	-Teach the limits of language and its role in suffering.<br>-Undermine cognitive fusion, using the passengers on the bus metaphor.
	Self as context	-Identify the self as the context in which inner experiences occur, using the chessboard metaphor.<br>-Explain that the patient can't choose what inner experiences occur, but can choose what to do with them.
	Present-moment awareness	-Help the patient be present with inner experiences.<br>-Identify the importance of being present while not being heavily attached to inner experiences.
	Acceptance	-Identify opportunities for acceptance from out-of-session practise.<br>-Encourage acceptance of any problematic inner experiences.
	Committed action	-Make behavioural commitments to continue to reduce smoking.<br>-Make behavioural commitments to engage in values-based activities instead of attempting to control urges.
8-9	Values	-Define the concept of values.<br>-Clarify the patient's values and assess the consistency of his or her behaviour with those values, using the values assessment worksheet.
	Committed action	-Make behavioural commitments to continue to reduce smoking.<br>-Increase behavioural commitments to engage in value-based living, based on recent values work.<br>-Discuss relapse management using ACT skills.
10	Termination	-Review any processes that still need attention.

		-Summarize the treatment, using the Joe the Bum metaphor.<br>-Apply ACT processes to relapse management.<br>-Apply ACT processes to termination.<br>-Suggest a self-help workbook for continued progress.

Figure S1 Study flowchart

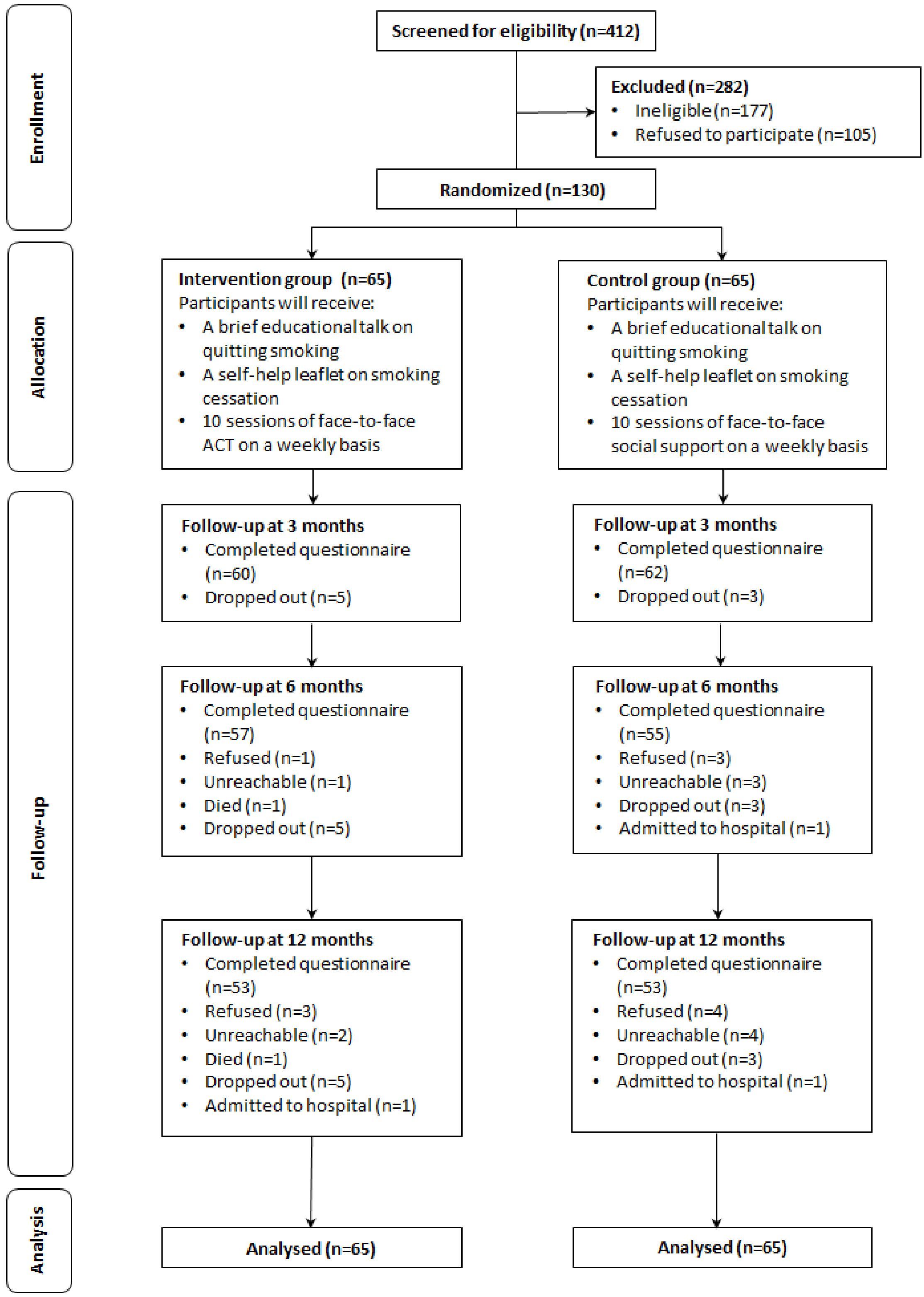

Supplement: Supplementary file 1 [file jcm-10-04304-s001.zip › jcm-1317904-supplementary.pdf]
